# Comorbid Opioid Use Disorder in Body Dysmorphic Disorder

**DOI:** 10.1192/j.eurpsy.2022.1182

**Published:** 2022-09-01

**Authors:** J. Kim, O. Alli-Balogun, G. Gill, P. Korenis, S. Mitra

**Affiliations:** 1 BronxCare Health System, Psychiatry, BRONX, United States of America; 2 Bronx Care health System, Psychiatry, Bronx, United States of America; 3 Bronx Care Health System-Affiliated with the Icahn School of Medicine at Mount Sinai, Psychiatry, New York, United States of America; 4 BronxCare Health System, Psychiatry, New York, United States of America

**Keywords:** opioid, Opioid Use Disorder, Body Dysmorphic Disorder

## Abstract

**Introduction:**

Body Dysmorphic Disorder (BDD) is a severe and common disorder that consists of distressing or impairing preoccupation with nonexistent or slight flaws in one’s physical appearance. People with BDD typically describe themselves as looking ugly, unattractive, deformed, or abnormal, whereas in reality they look normal or even very attractive.

**Objectives:**

Case Study

**Methods:**

Case Study

**Results:**

Mr. X is a 31 year-old male with history of Opiate (heroin, oxycodone) use disorder currently on maintenance (Buprenorphine-Naloxone) treatment. On admission, urine toxicology was positive for opiates and other drugs.CIWA score was 11. He was started on Lorazepam taper, Mirtazapine, Fluoxetine, and was started on Suboxone soon after. His cravings decreased and he was admitted for Rehab. He reports that anxiety associated with his “body image” related to ears, shape of head, eyebrows since he was in high school which made him “feel uncomfortable” going to school and concentrating in his classes. His coping mechanism was covering his head with hats, shaving eyebrows, substance use, and receiving an otoplasty.

**Conclusions:**

According to Houchins et al (2019), alcohol is the predominant substance used in BDD. It is interesting to note that only 6% of BDD patients had Opioid Use Disorder, but as this case demonstrates, can be a debilitating comorbidity that raises the risk for suicidality or hospitalization. However, little research has been done on the treatment of OUDs in patients with BDD or on the treatment of BDD in patients with an SUD, and this is an area of research that could benefit the modern population greatly.

**Disclosure:**

No significant relationships.

